# The role of the International Society for Stem Cell Research (ISSCR) guidelines in disease modeling

**DOI:** 10.1242/dmm.050947

**Published:** 2024-07-08

**Authors:** Cody Juguilon, Joseph C. Wu

**Affiliations:** ^1^ Stanford Cardiovascular Institute, Stanford University, Stanford, CA 94305, USA; ^2^ Department of Medicine (Division of Cardiovascular Medicine), Stanford University, Stanford, CA 94305, USA

**Keywords:** Disease modeling, ISSCR, Stem cells

## Abstract

Human stem cell-based modeling systems are valuable tools that can greatly improve the clinical translation of basic research. Importantly, the successful application of human stem cell-based models to biomedical research depends on the widespread adoption of ethical principles and practical standards. To achieve this outcome, the International Society for Stem Cell Research (ISSCR) provides a comprehensive set of recommendations that aim to promote the ethical usage of human stem cells and to ensure rigor and reproducibility within the field. Understanding and implementing these recommendations should be a top priority for investigators around the world.

## Teaser

**Summary:** The ISSCR provides comprehensive guidelines and standards for using human stem cells in biomedical research. Here, we discuss how and why these should be incorporated in disease modeling research.

## Introduction

Mounting competency within the field of stem cell biology has spawned a new age of biomedical research enabling sophisticated preclinical disease modeling using human-derived stem cells. In particular, human pluripotent stem cell (PSC) platforms represent a valuable tool for biomedical research, ranging from simple two-dimensional (2D) cultures of PSC-derived cell types to three-dimensional (3D) cultures containing multiple cell types (Liu et al., 2018). These systems are revolutionizing our ability to investigate disease mechanisms and promote the discovery of novel therapeutics while sidestepping the pitfalls of conventional animal-based research that often fails to translate to the clinic (Zushin et al., 2023). For example, PSC-derived cardiomyocytes have been successfully leveraged to elucidate patient-specific disease mechanisms, making them a valuable platform to investigate targeted therapeutics based on the patient's very own genetic background (Wang et al., 2023; Caudal et al., 2024). Moreover, biobanking is increasingly playing a crucial role in this process by significantly improving the accessibility of PSCs to researchers and industry, helping to create a powerful combined approach for conducting precise disease modeling and drug screening at scale, known as the ‘clinical trial in a dish’ (Sayed et al., 2020).

Although PSC technology holds great promise for the future of medicine, the ethical and practical aspects regarding the use of human stem cells require ongoing deliberation and renewed consensus as science progresses. To this end, the International Society for Stem Cell Research (ISSCR) has played a vital role in ensuring the efficient translation of basic research by putting forth Guidelines for Stem Cell Research and Clinical Translation (https://www.isscr.org/guidelines). These guidelines aim to ‘promote an ethical, practical, appropriate and sustainable enterprise for stem cell research and the development of cell therapies that will improve human health and should be available for patients in need’. To further address the practical considerations of human stem cell usage, the ISSCR also published the Standards for Human Stem Cell Use in Research (https://www.isscr.org/standards) in 2023, which emphasize practices that ensure rigor and reproducibility.

**Fig. 1.**
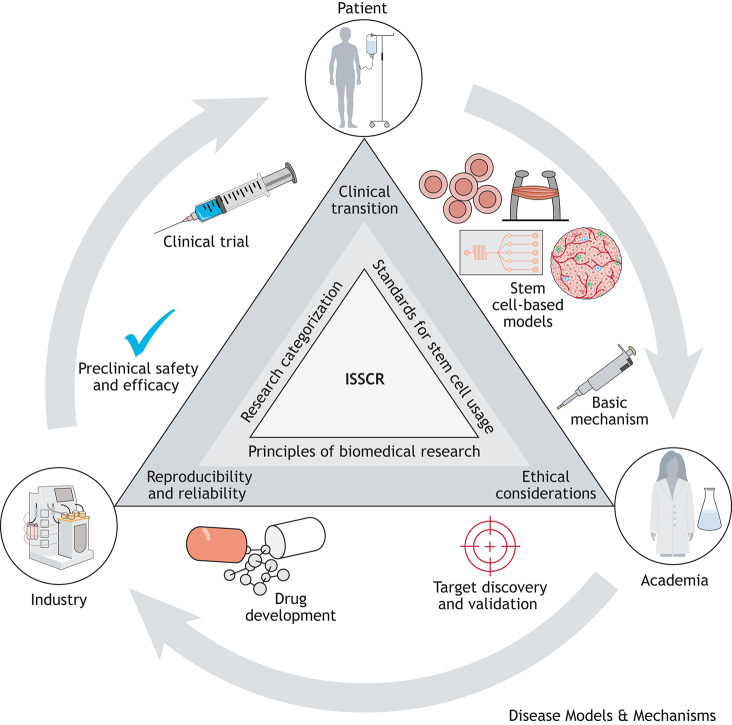
Vital role of the International Society for Stem Cell Research (ISSCR) in promoting the efficient translation of basic research.

Overall, the ISSCR guidelines encompass our ever-growing advancements in stem cell technology and application while maintaining a strong core of rigor, oversight and transparency. There is strong direct value in promulgating these guidelines in the disease modeling community, including adherence to fundamental ethical principles and requirements for research oversight. Additionally, a broader understanding of the standards and recommendations will encourage best practices in the usage of human stem cells and their derivatives to promote reliable and reproducible research outcomes required for efficient clinical translation (Fig. 1).

## Embodying the key principles of biomedical research

As researchers, we must recognize that the primary aim of biomedical research is to reduce human suffering resulting from disease and injury, which can only be achieved through efficient, rigorous and ethical practices. Thus, any successful scientific enterprise should embody a strong ethical backbone that prioritizes key principles, including scientific integrity, scientific transparency, emphasis on patient welfare, respect for research participants and fair distribution of the benefits of clinical translation to those in need. Together, these are the guiding features of the ISSCR that are particularly relevant during these times of rapidly evolving science and medical practice. Incorporating these key principles (detailed in Section 1 of the guidelines) at the individual/laboratory level will promote the efficient propagation of ethical science.

## Understanding the categorization of the use of human stem cells

Before initiating any research involving human embryos or stem cells, each investigator must understand where their research lies regarding the extent of review and oversight required. Recommendation 2.2 of the ISSCR guidelines provides categorizations of human embryo and stem cell usage, which essentially aims to ensure that due consideration and uniformity in practices are met in research and to specify scientific projects that should be subjected to a review process. The use of human embryos and stem cells can be categorized as follows:

**(1A)** Exempt from review by specialized oversight process
**(1B)** Reportable to oversight process but exempt from review
**(2)** Reviewed by specialized oversight process
**(3A)** Not allowed – currently unsafe
**(3B)** Not allowed – lacking scientific rationale or is ethically concerning

Examples of research activities that fall into category 1A include the transfer of human stem cells into postnatal animals, as well as most *in vitro* work with PSCs and organoid cultures. Research involving human embryos and stem cell-based embryo models can fall into categories 1B or 2, depending on the duration of culture and the extent of integration. A more detailed review of the 2021 updated guidelines can be found in Love-Badge et al. (2021).

## Ensuring reliability and reproducibility in stem cell research

Preclinical models are essential to biomedical research, as they provide evidence of safety and efficacy before early-phase human studies are initiated. The push to widely adopt the principle of the three Rs (Replacement, Reduction and Refinement) is exemplified by the recent enactment of the US Food and Drug Administration (FDA) Modernization Act 2.0, which permits therapeutics to enter clinical trials without the need for animal testing by using suitable ‘non-clinical tests’ (Moutinho, 2023). The success of this paradigm shift from animal usage to *in vitro* testing will rely heavily on human stem cell-based models, such as PSCs and their derivatives. However, a major limitation to the widespread adoption of these models is related to their reliability and reproducibility. For example, PSC-derived organoid models demonstrate significant heterogeneity and variability within an organoid (intra-organoid), among organoids in the same culture, and among organoids derived from individual patients (inter-organoid) (Zhao et al., 2022). Given the increasing complexities of our PSC-derived models, variability could be the result of any number of components such as differences in stem cell quality, extracellular matrix, medium or execution of a protocol. Thus, standardizing the entire process including human sample acquisition, stem cell characterization, model generation, experimentation and reporting should be a top priority.

For this reason, the ISSCR published the Standards for Human Stem Cell Use in Research to promote reproducibility and reliability within the field. This document provides a comprehensive description of the best practices for laboratory research with human stem cells with a focus on four major areas: basic cell characterization and maintenance, characterization of the undifferentiated state and assessing pluripotency, genomic characterization, and use of stem cell-based model systems. Importantly, these recommendations are aimed at establishing the minimum or baseline characterization and reporting measures that are feasible for any laboratory working with human stem cells (Ludwig et al., 2023).

## Biobanks as the gatekeepers for stem cell research and translation

Biobanks are expected to play a major role in the future of medicine by providing access to patient PSCs for research and drug development. The Standards for Human Stem Cell Use in Research document has details on the principles of biobanking, which will be a key aspect of ensuring cell quality as we increasingly rely on biobank services. Notably, the Stanford University Cardiovascular Institute (SCVI) Biobank exemplifies the principles outlined in the Standards for Human Stem Cell Use in Research and continues to compile a growing biorepository of deidentified human induced PSCs (iPSCs) from a wide range of cardiac diseases, using healthy and non-cardiac disease controls. The SCVI Biobank provides an extraordinary service to the scientific community by offering these iPSC lines to investigators for their research, a service that has promoted a multitude of scientific discoveries (Wei et al., 2022; Tu et al., 2024). In addition, many of the iPSC lines from the SCVI Biobank have been characterized per the Standards for Human Stem Cell Use in Research and are available for use by researchers (Melesio et al., 2024).

## Conclusion

As we embark on the next frontier of biomedical innovation with a greater focus on ensuring efficient translation from basic science to patient care, we must keep in mind relevant ethical and practical considerations of using human stem cells and their derivatives. Stem cell-based modeling systems can change medicine as we know it, potentiating wondrous scientific discoveries and therapeutic applications, while reducing the need for animal testing. The ISSCR guidelines and standards present an important set of recommendations that will ensure the successful propagation of scientific discovery by promoting ethical and practical standards that result in robust, reproducible and reliable results, a cornerstone of any successful translatable scientific enterprise. Taken together, these guidelines and standards represent a foundation that the Disease Models & Mechanisms community should consult with and incorporate into their daily research practices.
